# Light microscopic evidence of in vivo differentiation from the transplanted inferior turbinate-derived stem cell into the rod photoreceptor in degenerating retina of the mouse

**DOI:** 10.1186/s42649-020-00031-w

**Published:** 2020-06-03

**Authors:** Yong Soo Park, Yeonji Kim, Sung Won Kim, In-Beom Kim

**Affiliations:** 1 Department of Anatomy, College of Medicine, The Catholic University of Korea, 222 Banpo-daero, Seocho-gu, Seoul, 06591 South Korea; 2 Catholic Neuroscience Institute, College of Medicine, The Catholic University of Korea, 222 Banpo-daero, Seocho-gu, Seoul, 06591 South Korea; 3 Department of Otolaryngology-Head and Neck Surgery, Seoul St Mary’s Hospital, College of Medicine, The Catholic University of Korea, 222 Banpo-daero, Seocho-gu, Seoul, 06591 South Korea; 4 Catholic Institute for Applied Anatomy, College of Medicine, The Catholic University of Korea, 222 Banpo-daero, Seocho-gu, Seoul, 06591 South Korea

**Keywords:** Human turbinate-derived mesenchymal stem cell, Rod photoreceptor, Retinal degeneration, Confocal microscope, Differential interference contrast

## Abstract

The human turbinate-derived mesenchymal stem cells (hTMSCs), which were DiI-labeled and transplanted into the subretinal space in degenerating mouse retina, were observed in retinal vertical sections processed for rhodopsin (a marker for rod photoreceptor) by confocal microscope with differential interference contrast (DIC) filters. The images clearly demonstrated that DiI-labeled hTMSCs have rhodopsin-immunoreactive appendages, indicating differentiation of transplanted hTMSC into rod photoreceptor. Conclusively, the finding suggests therapeutic potential of hTMSCs in retinal degeneration.

Retinal degeneration (RD) is a various group of diseases, such as age-related macular degeneration (AMD), retinitis pigmentosa (RP), and Stargardt disease, characterized by the irreversible and progressive degeneration of photoreceptor cells in the retina, resulting in blindness (Rattner and Nathans, 2006). Because the retina belongs to the central nervous system, including brain and spinal cord, there are few clinical treatments and little recovery from blindness. Recently, stem cell therapy, photoreceptor replacement by transplantation of stem cells is proposed as an important treatment strategy for RD (Pearson, 2014; Blau and Daley, 2019).

For the successful photoreceptor replacement therapy, a key factor is the donor cell that has an ability of differentiation into the photoreceptor and migration/integration into the laminar structure of the retina. In this study, we introduced human turbinate-derived mesenchymal stem cells (hTMSCs) as a candidate of stem cell therapy (Hwang et al., 2012; Hwang et al., 2014) for retinal degeneration, which cells showed multipotent MSC with therapeutic potential for acute stroke (Lim et al., 2018).

One micro-liter (1 × 10^6^/100 μl) of DiI-labeled hTMSC suspension was injected to the subretinal space in BALB/c mouse, in which RD was induced by exposed to the 2000 lx of blue-LED for 2 h. After 14 days from injection, eyecups were prepared, fixed in 4% paraformaldehyde, and embedded for frozen section. Retinal vertical sections were immuno-stained by rhodopsin and opsin which are known as rod and cone photoreceptor marker, respectively. The sections were observed by using confocal microscope (LSM 800 with Airyscan; Carl Zeiss Co. Ltd., Oberkochen, Germany) with differential interference contrast (DIC) filters.

Expression of the rhodopsin and opsin in the injected cells was shown in Fig. 1. DiI-labed hTMSCs (red) were found in the subretinal space. Rhodopsin (white, Fig. 1a) was mainly expressed in the outer segment of the photoreceptor, and opsin (green, Fig. 1b) was expressed in the cone photoreceptor cells. Because mice are rod-dominant animal, most of photoreceptors expressed rhodopsin rather than opsin. In higher magnification images, rhodopsin was localized around the cell body of the injected hTMSCs (Fig. 1c), while opsin was not detected within the cells (Fig. 1d). To know whether the rhodopsin is expressed in the injected cells or it is separated particle from segment layer of the retina, we obtained DIC images (Fig. 1e). In a merged image with DIC one, these rhodopsin-labeled puncta (arrows in Fig. 1e) appeared to be bulging appendages of the injected hTMSCs. The result indicates that hTMSCs may differentiate into the rod photoreceptor in degenerating retina. Conclusively, it suggests that hTMSCs are a strong candidate for the stem cell therapy for retinal degeneration.

**Fig. 1:**
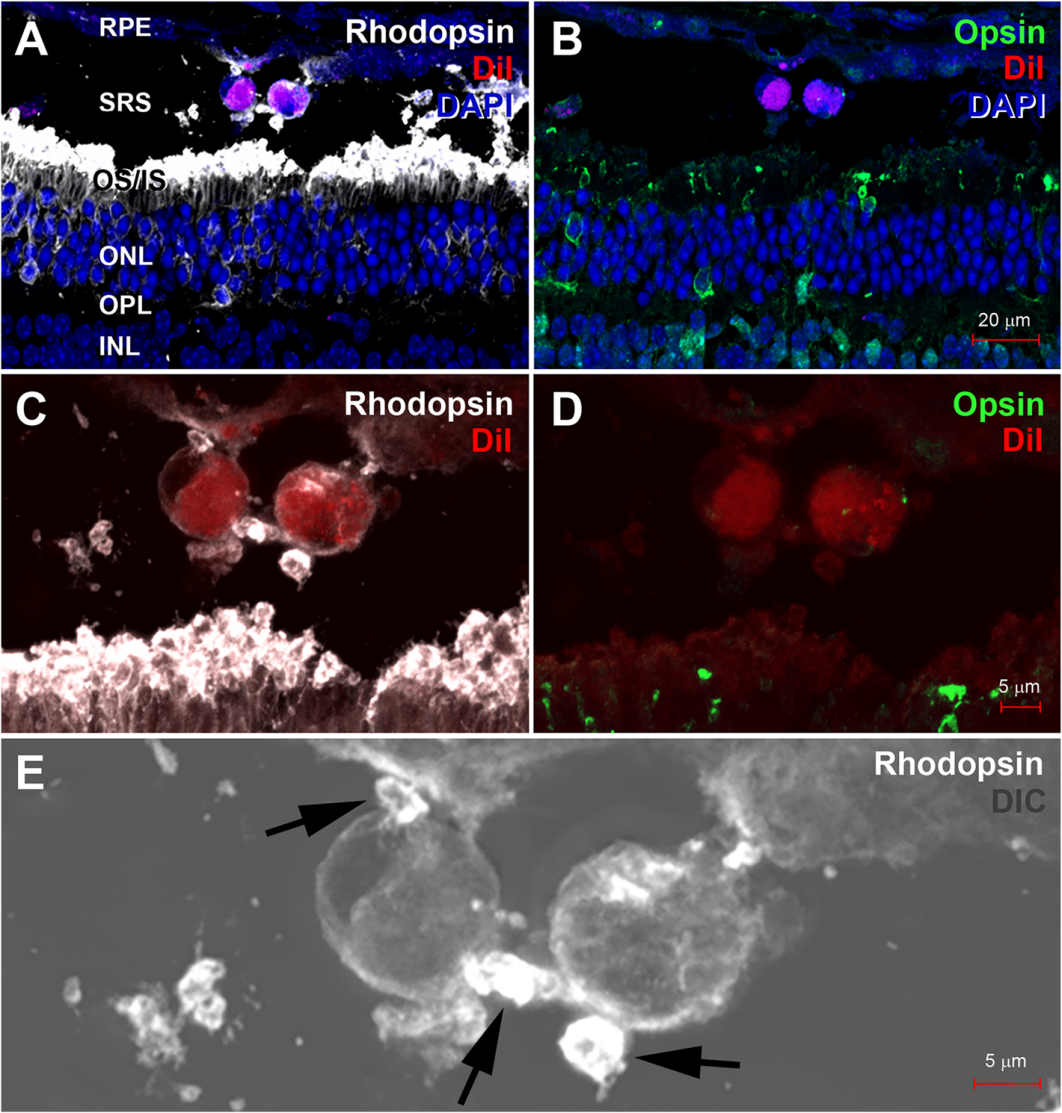
**a, b** DiI-labeled hTMSCs (red) were observed in the subretinal space (SRS) of the retina. In A, rhodopsin (white) was expressed in the outer segment of the rod photoreceptor in outer and inner segments layer (OS/IS) and near two injected hTMSCs. In **b**, Opsin (green) was expressed in the cone photoreceptors. However, hTMSCs (red) did not express opsin. DAPI was counterstained for nuclei of the retina. RPE, retinal pigment epithelium; ONL, outer nuclear layer; OPL, outer plexiform layer; INL, inner nuclear layer. **c**, **d**. C and D were magnified from A and B, respectively. A few rhodopsin-labeled puncta (white in C) are placed close to the hTMSCs (red in C), while opsin-labeled puncta (green in D) are absent around the hTMSCs (red in D). **e** In this higher magnified merged image of DIC and confocal image showing rhodopsin immunoreactivity, three rhodopsin-labeled puncta (arrows) appears to be bulging appendages of two DiI-labeled hTMSCs (red)

## Data Availability

Not applicable. “Please contact the corresponding author for data requests.”
